# Impact of perinatal environmental health education intervention on exposure to endocrine disruptors during pregnancy—PREVED study: study protocol for a randomized controlled trial

**DOI:** 10.1186/s13063-021-05813-5

**Published:** 2021-12-04

**Authors:** Houria El. Ouazzani, Steeve Rouillon, Nicolas Venisse, Lynda Sifer-Rivière, Antoine Dupuis, Guillaume Cambien, Sarah Ayraud-Thevenot, Anne-Sophie Gourgues, Pascale Pierre-Eugène, Fabrice Pierre, Sylvie Rabouan, DisProSE Group, Virginie Migeot, Marion Albouy-Llaty

**Affiliations:** 1 Health-Endocrine Disruptors-EXposome (HEDEX), INSERM-CIC1402, University Hospital of Poitiers, 2 rue de la Milétrie, 86021 Poitiers CEDEX, France; 2 Faculty of Medicine and Pharmacy, University of Poitiers, 6 rue de la Milétrie, 86000 Poitiers, France; 3 BioSPharm Pole, University Hospital of Poitiers, 2 rue de la Milétrie, 86021 Poitiers CEDEX, France; 4 UMR CNRS 7285, IC2MP, Poitiers, France; 5 Research Center of Medicine, Sciences, Health and Society (Cermes 3), EHESS, University of Paris Descartes, Villejuif, France; 6 Department of Obstetrics and Gynecology and Reproductive Medicine, University Hospital of Poitiers, 2 rue de la Milétrie, 86021 Poitiers CEDEX, France

**Keywords:** Endocrine disruptors, Maternal exposure, Pregnancy, Environmental health, Intervention research, Randomized controlled trial

## Abstract

**Background:**

The suspected or actual effects on health of endocrine-disrupting chemicals (EDC) and their ubiquitous presence in everyday life justify the implementation of health promotion interventions. These interventions should ideally be applied during critical windows like pregnancy. Perinatal environmental health education interventions may help to reduce EDC exposure during pregnancy.

**Methods/design:**

PREVED (Pregnancy, PreVention, Endocrine Disruptors) is an open-label randomized controlled trial assessing the impact of environmental health education intervention on EDC exposure during pregnancy. Inclusion, consent, and randomization take place during the first trimester. The participants are randomly allocated into three groups: (i) control group (information leaflet on EDCs), (ii) intervention group in neutral location (information leaflet and workshops in a meeting room), and (iii) intervention group in contextualized location (information leaflet and workshops in a real apartment). Workshops are organized between the second and third trimesters of pregnancy. Main outcome is the percentage of participants who reported consuming manufactured/industrial food. Secondary outcomes are as follows: (i) psycho-social dimensions, (ii) EDC concentrations in urine, (iii) EDC concentration in colostrum, and (iv) percentage of participants who reported consuming paraben-free personal care products.

**Discussion:**

PREVED is a ground-breaking intervention research project dedicated to perinatal environmental health education that aims to identify pollutant sources in daily life and to offer accessible and realistic alternative solutions, by promoting the sharing of know-how and experience in a positive and non-alarmist approach.

**Trial registration:**

ClinicalTrials.gov: NCT03233984 (current status: ongoing). Retrospectively registered on 31 July 2017 (https://clinicaltrials.gov/ct2/show/NCT03233984) because when the first participant was enrolled in this non-drug intervention, ClinicalTrials.gov was centered in therapeutic trials.

The World Health Organization Trial Registration Data Set is in Additional file 1.

**Supplementary Information:**

The online version contains supplementary material available at 10.1186/s13063-021-05813-5.

## Background

The in utero period is an important period that conditions health in adult life. Prenatal exposure can disturb fetal and neonatal development [1] and lead to numerous disorders [2–4]. These health consequences are especially underlined by FOAD- (Fetal Origins of Adult Disease) [5], DOHaD- (Developmental Origins of Health and Disease) theories [6, 7], which emphasize the importance of intrauterine environmental exposure in offspring health, and by POHaD-theory (Paternal Origins of Health and Disease), which underlines the role of paternal environment and exposure [8].

The concept of “exposome” initiated in the 2000s is along the same lines. Indeed, exposome includes lifetime exposures from periconceptional period until death [9]. Under this concept, exposures can be classified as [10, 11]:

- Internal exposure: combining all endogenous factors
- Specific external exposure: combining exogenous factors such as chemical contaminants, environmental pollutants and lifestyle factors
- General external exposure: taking into account a wider environment with social, economic, and psychological factors

Among these exposure factors, endocrine-disrupting chemicals (EDCs) are chemicals and environmental pollutants defined by the World Health Organization (WHO) as “exogenous substances or mixture that alter function(s) of the endocrine system and consequently cause adverse health effects in an intact organism, or its progeny, or (sub)-populations” [12]. EDCs may be synthetic such as parabens (PB) and phthalates found in cosmetics, bisphenol A (BPA) found in plastics, or pesticides such as atrazine or trifluralin found in soil or food. They may also be natural such as hormones or phytoestrogens [13].

Their involvement is confirmed, or at least suspected, in the development of numerous diseases or disorders, especially on reproduction, hormone-dependent cancers [14, 15], and metabolic disorders [16]. This is typically the case of diethylstilbestrol, an estrogen medication which was prescribed for pregnant women in the 1940s [17]. Many decades later, prenatal exposure to diethylstilbestrol was recognized as the cause of many diseases for children exposed in utero, and even for grandchildren who had never been directly exposed [18–20]. In fact, exposure to several EDCs at specific developmental stages called “susceptibility” or “critical windows” such as prenatal and puberty periods causes more pronounced health effects [14, 21, 22].

In 2016, the European Commission made recommendations on prioritizing human epidemiology (follow-up cohorts…), knowledge about exposure (chemical markers, biomarkers…), and effects in wildlife [23], and in its strategic approach, issued in 2018, emphasis was put on prevention interventions, particularly in “critical windows” [24]. Thus, several steps were necessary to take into account EDC exposure [25].

Promotion health interventions should be carried out since the pre/periconceptional period [26], which comprises key moments of exposure sensitization and environmental health promotion [27, 28]. There are numerous recommendations and interventions aimed at improving maternal and infant health, for instance, on nutrition [29–31], iron supplementation [32], physical activity [33], hypertensive disorders [34], oral health [29], or on reduction of environmental tobacco smoke exposure [35, 36]. However, only a few of them concern EDC exposure [37–40].

To our knowledge, there exists no research study focused on perinatal exposure through the prism of environmental health education in France. Our hypothesis was that an extended perinatal environmental health education intervention conducted during pregnancy by promoting the sharing of know-how and experience in a positive and non-alarmist approach would contribute to the reduction of EDC exposure.

The PREVED (Pregnancy, PreVention, Endocrine Disruptors) study aimed to assess the impact of an environmental health education intervention on prenatal exposure to EDCs. The secondary objectives are to assess the impact of the intervention on psycho-social dimensions, EDC concentration in urine and colostrum, and on the choice of personal care products. The EDCs studied are BPA, its chlorinated derivatives, and PB: methyl-, ethyl-, propyl-, and butyl-PB.

## Methods

### Study design

The PREVED study is an ongoing open-label, monocentric, randomized (1:1:1) controlled superiority trial, parallel-designed, three-armed with:

- A control group: group (1)
- An intervention group in neutral location: group (2)
- An intervention group in contextualized location: group (3)

### Participants and interventions

The PREVED study is conducted in Poitiers (France). Recruitment was held from April 2017 to April 2019. The site of intervention is in an underprivileged and multicultural Poitiers neighborhood with strong associative potential (animation center, young workers’ home and sports center), where women of low socioeconomic and educational status may be particularly exposed to EDCs [41].

According to the allocation group, workshops are performed in two different locations. Group 2’s intervention is performed in a meeting room in the “Animation Centre of the neighborhood,” which is a neutral location. Group 3’s intervention is performed in “*Atelier du 19*,” an apartment dedicated to health/environment education in everyday life [42].

Inclusion and exclusion criteria are detailed in Table 1.

**Table 1:** Inclusion and exclusion criteria for the PREVED study

Inclusion criteria	Exclusion criteria
- Pregnant women with declared pregnancy - Speaking French - Being aged 18 years or more - Living in the French department of Vienne and at less than 30 min from Poitiers - Having the intention to give birth in the maternity of the University Hospital, of the clinic “Fief de Grimoire” in Poitiers or in the Hospital of Châtellerault - Having signed a consent form	- Pregnant women expecting twins or more - Having a complicated pregnancy - Not speaking French - Being under 18 years old or under legal protection despite being 18 or more - Being deprived of liberty by judicial or administrative decision - Undergoing psychiatric treatment - Not being affiliated to a social security system - Intending to move out during the next year - Having the intention to give birth in a maternity ward other than the department of Vienne - Being unable to express consent

PREVED study implementation was preceded by an essential step involving pregnant women and professionals. Qualitative and quantitative studies were carried out to describe pregnant women’s knowledge, attitudes, and behaviors towards EDC exposure [43], to estimate their risk perception [44], and to determine environmental health knowledge, attitudes, and practices of prenatal healthcare providers [45]. These results facilitated the design of the intervention and the identification of the aims and outcomes of PREVED study.

Furthermore, the study was preceded by the creation of a “DisProSE Group” steering committee, a consortium of researchers, local actors, and decision-makers who co-built the intervention, initially developed by a mutual insurance company, according to PREVED’s aims and to behavior change techniques (BCT) taxonomy [46].

After complete recruitment, we could analyze the intervention, which involved 12 of the 16 BCTs, in view of diversifying approaches (Table 2).

**Table 2:** Behavior change techniques (BCT) used for the intervention of PREVED study

Grouping of BCT taxonomy	Chosen BCT in each BCT group	Example of an intervention
*Goals and planning*	Problem solving	- To identify behaviours that are easy to implement daily to reduce exposure to endocrine disruptors chemicals (EDC) (example: replace plastic with glass)
	Action planning	- To integrate the purchase of primary products into your personal planning and devote time to manufacturing cosmetics at home
*Feedback and monitoring*	Not applicable	- No feedback between the workshops/Only a series of workshops by participant
*Social support*	Not applicable	- Few attendants came to the workshops
*Shaping knowledge*	Training on how to perform the behavior	- To learn to read package labels - To learn to prepare cookies and cosmetics at home
	Behavioral experiments	- To appreciate the changes implemented daily to reduce their exposure to EDCs thanks to the three-time questionnaire which to compare her consumption before and after
*Natural consequences*	Information about health consequences	- To respond to participants' questions about the known effects of EDCs exposure during pregnancy
	Information about social and environmental consequences	- To present the consequences of behaviours aimed at reducing EDCs exposure on the environment (example: waste reduction through homemade products)
*Comparison of behavior*	Demonstration of the behavior	- To teach how to make one’s own cosmetics
*Associations*	Prompts/clues	- To highlight leaflets and documentation at home
*Repetition and substitution*	Behavioral practice/rehearsal	- To integrate the manufacture of cosmetics into your schedule: the recipes offered were inexpensive and easy to integrate into a routine without constraints
	Behavior substitution	- To avoid heating food in plastic dishes with a microwave oven
	Habit formation	- To encourage participants to ventilate the house for 20 minutes/day
	Habit reversal	- To use glass jars and boxes to store food and meal leftovers, instead of plastic packaging
	Generalization of target behavior	- To teach how to make one’s own cosmetics in order to be independent/repeat the manufacture at home
	Graded tasks	- “Change everything” is neither taught nor required to propose simple solutions to reduce exposure to EDC
*Comparison of outcomes*	Credible source	- To use of current data from literature
	Pros and cons	- To explain that using glass packaging instead of plastic is valuable but not without some constraints (heavier, risk of breaking)
	Comparative imagining of future outcomes	- To project yourself at home (eliminating / reducing EDCs sources) in particular to safe your health and your children health
*Reward and threat*	Material reward	- To bring home the products made during the workshops (floor cleaner, cookies, ...): quick practice and experimentation directly after the workshop - To reimburse the travel costs of participants (planned but not implemented)
*Regulation*	Conserving mental resources	- To recall the pleasure of consuming healthy products - To encourage the implementation of even small changes on a daily basis: Choose not-guilty and counterproductive words
*Antecedents*	Adding objects to the environment	- To bring home made products: reusable containers will remind the participants of the value of making their own products and encourage them to continue manufacturing
*Identity*	Framing/reframing	- To rely on a presentation of a positive vision of health - Do not focus on pathologies - To encourage exchanges: sharing of experiences and knowledge is complementary to the information given by the facilitators
	Incompatible beliefs	- To remember that a "Natural Product" is not necessarily a "Safe Product" or a "Healthy Product" - To explain that it is not necessary to go from “all industrial” to “all homemade”: by reading the labels, it is possible to better choose products containing little or no EDCs (number of ingredients, absence of 'additives ...)
*Scheduled consequences*	Not applicable	
*Self-belief*	Verbal persuasion about capability	- To reassure participants: no guilty speech - To highlight simple and accessible solutions to limit EDCs exposure: positive reinforcement
	Focus on past success	- To encourage the sharing of experience and information between participants (example: multiparous participants can advise new parents on "tips and tricks")
*Covert learning*	Not applicable	

No concomitant intervention is prohibited. Women randomized in the control group are told that they are entitled to have the program after the end of the study (1 year after delivery).

The list of pregnant women is obtained through pregnancy declarations, which are centralized in rance by “*Protection Maternelle et Infantile*” (maternal and child protection) or PMI. The investigator sends to eligible women an informative postal mail with a prepaid envelope. Women who wished to participate in PREVED study confirmed their interest by phone or mail.

To improve enrolment, information leaflets are sent to general practitioners and midwives working in the enrolment area. To achieve representative and adequate enrolment, PMI’s midwives were trained to present the PREVED study to pregnant women. Local actors in social centers and the associative sector also assisted in enrolment. The first participant was enrolled on 17 April 2017.

The intervention consisted of a sequence of three workshops held between the second and the third trimesters of pregnancy. It incorporated the results of a previous workshop [43] and aimed to identify pollutant sources in daily life and to offer accessible and pragmatic alternative solutions by promoting the sharing of know-how and experience in a positive and non-alarmist approach. Three themes are addressed (Table 3). Any participant may at any time withdraw from this study and adherence participation is encouraged with adapted meeting.

**Table 3:** Detailed workshops for the intervention of PREVED study

	Workshop 1	Workshop 2	Workshop 3
***Theme***	Indoor air quality	Nutrition	Personal care products
***Duration***	2 h	2 h	2 h
***Participants***	10 people: pregnant women ± spouse, friend, or parent	10 people: pregnant women ± spouse, friend, or parent	10 people: pregnant women ± spouse, friend, or parent
***Facilitator***	Medical advisor for indoor environments	Dietician trained in environmental health	Cosmetologist
***Pedagogic objectives***	- To detect and reduce sources of domestic air pollution - To share know-how, experiences, and information on alternatives	- To identify food pollutants - To share know-how, experiences, and information on alternatives	- To identify necessary elements to make healthy choices of personal care products and clothes - To share know-how, experiences, and information on alternatives

The DisProSE group produced an information leaflet providing advice on ways of reducing EDC exposure in relation to the aforementioned themes. This leaflet was designed according to the principles of health literacy to be as accessible and comprehensible as possible [47]. All participants, including group 1, receive it during the first home visit. The only difference between group 2’s workshops and group 3’s workshops is the location of the intervention.

**Fig. 1:**
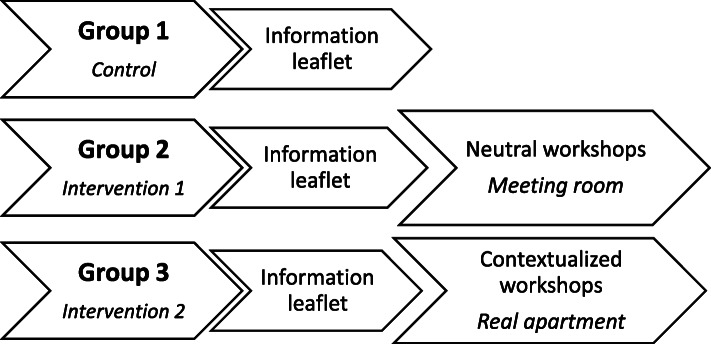
Description of the three groups of the PREVED study

Fig 1. summarizes the conduct of PREVED study.

#### Main outcome

- Percentage of participants who reported consuming manufactured or industrial food determined through a sociodemographic and consumption questionnaire (Q1) developed by our research team [48] and administered at t0, t + 2 months and t + 14 months.

#### Secondary outcomes

- Mean score of psychosocial questionnaire (Q2) developed by our team, whose dimensions are presented in Table 4
- Urinary concentrations of: BPA, chlorinated derivatives of BPA, methyl-, ethyl-, propyl-, and butyl-PB
- Concentration in colostrum of: BPA, methyl-, ethyl-, propyl-, and butyl-PB
- Percentage of participants who reported consuming PB-free personal care products

**Table 4:** Dimensions of the psychosocial questionnaire used in PREVED study

Dimension of the psychosocial questionnaire	Origin of corresponding question or questions	Comment
***Self-esteem***	French version of the “Self-esteem scale” [49]	Score ranges from 10 to 40: the lower the score, the lower self-esteem. Items for this score are self-administered
***Perceived health***	Created for the questionnaire	Score ranges from 0 to 100 on a visual analogic scale
***Health care renunciation and risk aversion***	Inspired by French national investigations [50, 51]	Use of visual analogic scales
***Risk perception***	Created for the questionnaire and based on the Perception of Pregnancy Risk Questionnaire [52]	Use of visual analogic scales. A composite and global score of perinatal risk perception related to EDC exposure was created
***Knowledge about endocrine-disrupting chemicals (EDCs):*** routes and sources of exposure, ability to name some EDC’s molecules or families of molecules and definition of an EDC	Created for the questionnaire	A composite score was created. A catalog of photo images illustrates sources of exposure
***Perceived knowledge about EDCs***	Created for the questionnaire	Use of a visual analogic scale
***Expectations for a “healthy baby”***	Based on “the healthy baby concept” [53]	Use of a visual analogic scale and a catalog of photo images
***Trusted person***	Created for the questionnaire	The answer to this question aims to identify the trusted person, who exerts the most influence on the pregnant woman
***Level of concern about five risks related to pregnancy***	Created for the questionnaire	This exploration is meant to establish a hierarchy, with respect to chemical risk concerns. A catalog of photo images is used
***The way the pregnant woman heard about EDCs and, if so, how she experienced the information she received***	Created for the questionnaire	These two parameters explore the role of information provided by the media, professional studies, and relationships (personal, friendly, professional, health professionals)
***Relationship to risk visibility***	Created for the questionnaire	Likert-type response

**Fig. 2:**
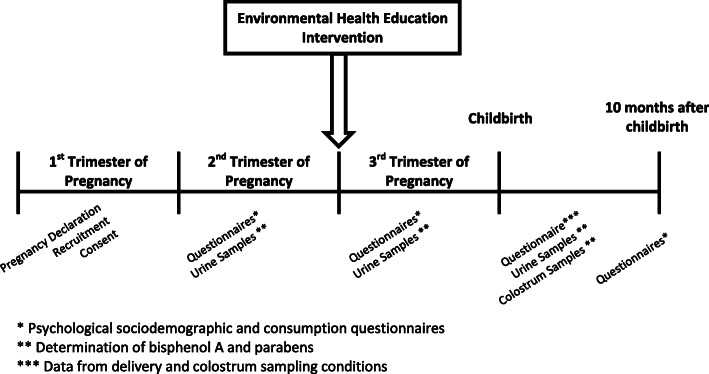
Course of the PREVED study

The planning of the administration of questionnaires and the collection of samples is summarized in Fig 2.

Time schedule of enrolment, intervention, assessments, and visits are detailed in Table 5. They were carried out according to the SPIRIT Guideline (Additional file 2).

**Table 5:** SPIRIT flow diagram of schedule of enrolment, interventions, and assessments in PREVED study

	Enrolment	Post-allocation				Close-out	
Timepoint	***Baseline***	***t***_***0***_	***t***_***+1M***_	***t***_***+2M***_	***t***_***+4M***_	***t***_***+14M***_	
		Home visit 1		Home visit 2	Childbirth	Home visit 3	
		Leaflet	Workshops				
**Enrolment:**							
**Eligibility screen**	X						
**Informed consent**	X						
**Allocation**	X						
**Interventions:**							
***Group 1*** *Control group*		X					
***Group 2*** *Intervention group in neutral location*		X	X				
***Group3*** *Intervention group in contextualized location*		X	X				
**ASSESSMENTS:**							
***Q1*** *Sociodemographic questionnaire + consumption habits*		X		X		X	
***Q2*** *Psychosocial questionnaire*		X		X		X	
***Collection of urine samples***		X		X	X	X	
***Colostrum samples***					X		

### Sample size

According to the results of the EDDS (Endocrine Disruptors Deux-Sèvres) cohort study, 83% of French women consumed canned food [48]. Thus, we calculated the number of participants based on the primary outcome measure (consumption measure) using a two-sided paired sample *t* test with 0.05 level of significance two-way design and *β* = 0.20. Our hypothesis was that the contextualized intervention would decrease this percentage to 60%, representing a decrease of 23 points, and 58 participants were required for each group. We expected 20% of lost to follow-up participants; consequently, 70 participants are required for each group, out of a total of 210 pregnant women to be included in our study. This number was increased to 273 persons due to significant colostrum loss in an amendment submitted to the Committee for Personal Protection who approved the first protocol (protocol version 10 approved by the Committee for Personal Protection on 15 June 2018).

### Assignment of interventions

The trial is open-labeled but a central random blind-generated allocation was performed before the first home visit. The methodological referent of PREVED study enrolled the participants. A number was assigned to each participant. The random blind-generated allocation with 1:1:1 ratio is based on fixed blocks of 3 before the first home visit. The allocation sequence is generated on Microsoft EXCEL® (function RAND) by a doctoral student. Participants were then randomly assigned to one of the three groups by a research nurse who is also trained in indoor environments.

This type of study design did not allow a blinded trial as is the case in intervention research.

No changes were planned to randomly assigned groups.

### Data collection, management, and analysis

The research nurse performs home visits and collected data at four key moments (Table 5):

- Home visit 1: Q1, Q2, and urine analysis
- Home visit 2: Q1, Q2, and urine analysis
- Childbirth: childbirth data, urine, and colostrum analysis
- Home visit 3: Q1 and Q2

Data input is entered by the research nurse and by the study site coordinator on electronic case report forms (e-CRF). Quality control and queries are carried out regularly. Telephone reminders are expected, if necessary, for home visits 2 and 3.

Urine and colostrum samples are collected in glassware provided by the investigator. They are stored at − 80 °C, in polypropylene tubes, in the Biological Resource Centre of the University Hospital of Poitiers (no. BB-0033-00068) for 5 years. These samples are analyzed using reliable ultrasensitive methods previously developed by our research team for BPA and its chlorinated derivatives [54, 55] and new methods for PB developed for this study. All laboratory materials and solvents are tested to ensure that they were free of contamination from target compounds. High-quality compounds and solvents are supplied by reagent manufacturers. Samples are prepared and extracted. Analyte concentrations are determined using Ultra Performance Liquid Chromatography (Shimadzu®, Kyoto, Japan) coupled to an API 6500+ mass spectrometer (ABSciex®, Concord, Canada) equipped with an electrospray ionization (ESI) interface, operating in negative ionization mode. Target analytes are analyzed in multiple-reaction monitoring (MRM) mode using two specific transitions per analyte to ensure the selectivity and the sensitivity of the method. The analytical methods used are validated according to international guidelines [56–59]. These methods allow to detect and quantify trace concentrations of unconjugated forms of BPA, its chlorinated derivatives, and PB. Urinary concentrations of analytes are corrected using urinary creatinine concentrations determined with a Cobas® 8000 (Roche Diagnostics, Mannheim, Germany) and using urinary specific gravity determined with a hand-held refractometer.

Firstly, a descriptive analysis will be performed on sociodemographic data, questionnaire scores, and on EDC concentrations with percentage for qualitative variables and mean, standard deviation, median, maximum, and minimum for quantitative variables.

Baseline measurements of the three trial arms will be compared using paired *t* tests and *χ*^2^ tests for the analysis of Q1 and EDC concentrations. The results of Q2 in the three groups will be compared by dimension using ANOVA analyses. An intention-to-treat analysis will carried out, and then a per-protocol analysis in aim to take into account population relating to protocol non-adherence.

Main outcome will be compared between home visit 1 and home visit 2. The rest of analysis will be performed between home visit 1, home visit 2, and home visit 3. Multivariate logistic regression will be performed to assess factors influencing main and secondary outcomes.

All statistical analyses will be performed using SAS 9.4® (SAS Institute, Cary, North Carolina, USA).

No interim analyses are scheduled.

### Monitoring

A data monitoring committee was composed by a team from the Research Directorate of University Hospital of Poitiers. It is in charge of auditing trial conduct every 4 months, data management and promoting data quality. It consists of a Clinical Research Associate, a project monitoring manager, a data manager, and also the clinical trials vigilance unit. Consistency tests were performed on e-CRFs. As the study carries no risk, only consent and reporting of serious adverse events are verified.

The only expected adverse event was anxiety. Adverse events are noted at each home visit, throughout the period of participation of pregnant women.

## Discussion

To our knowledge, PREVED is the first study to assess the impact of perinatal environmental health education by promoting the sharing of know-how and experience in a positive and non-alarmist approach. Along with the randomized controlled trial, a sociological approach is applied to analyze the conditions of reach, efficacy, adoption, implementation, and maintenance of the intervention [60]. In particular, observations are carried out during the workshops using grids and describing the interactions between the pregnant women and the workshop animator [61]. Moreover, interviews with stakeholders (researchers, local actors and decision-makers) are conducted to evaluate the transferability of the intervention [62]. The collective and local dynamics underlying the development of intervention and the points of view of participants are thereby assessed [61].

The PREVED study adopted two complementary approaches in the framework of the intervention research process. In fact, intervention research applied to the health field is a specific kind of research that aims to provide information on the impact of interventions on population health using scientific methods [63] and to determine how “to intervene” (not “to discover”) [64]. This design should be well-founded and include partnerships with practitioners [65]. Thus, DisProSE group facilitated partnership with different stakeholders through an interdisciplinary approach.

The PREVED intervention takes on the form of practical 2-h workshops. This configuration is easily reproducible and viable. Indeed, in addition to mention of the effectiveness of intervention research, degree of viability and specification of the mechanisms explaining the effects should be considered in conjunction with “the interventional system” [66].

Furthermore, the main feature of PREVED study is to consider all aspects of lifestyle and to underscore key functions such as sharing of know-how, sharing of experiences and information between participants. In fact, few environmental promotion interventions have shown that EDC exposure was reduced by changing lifestyle. However, previous studies assessed the impact by taking into account only one component of lifestyle, such as nutrition or cosmetics [67, 68] and did not include the dimension of “know-how” [69]. Moreover, these studies took EDC concentrations in urine as their main outcome, even though analysis of the latter may be unreliable [70–72] and did not provide information about psychosocial aspects. This facet will be explored by the psychosocial questionnaire Q2 in our study. Concerning assessment of exposure to EDCs, we chose urine matrix samples because spot samples of urines are not restrictive and ensure optimal acceptability for the pregnant women participating. Twenty-four-hour urine collection may have been preferable, due to non-influence of the time of day. However, spot samples of urines provide reasonably reliable information on impregnation by BPA and PB during pregnancy [73–76].

We made sustained efforts to minimize information bias. Notwithstanding the absence of blindness, the investigator’s practices and behaviors are likely to vary during visits, according to allocation group. That is why hetero administration of questionnaires by a single trained assessor, with a standardized interviewer’s guide, has been adopted. Furthermore, selection bias is controlled by trained PMI nurses wishing to facilitate the enrolment of pregnant women facing difficulties. Enrolment bias is thereby avoided, whereas preceding studies highlighted the fact that participants for this type of study are mostly women with a high socio-economic level [48]. However, in such a cohort, selection bias can still be expected [77] especially for pregnancy cohorts, insofar as women with a high socio-educational level are likely to feel more concerned [78, 79]. We will probably need to determine how to reach populations more effectively in precarious situations. At the time of the analysis, results will be adjusted on confounding factors that affect the psycho-social dimensions and have been highlighted in this study.

## Conclusion

Through this study, we aspire to increase all health stakeholders’ awareness of the importance of primary prevention on exposome, especially EDC exposure, during pregnancy. In fact, given the methodological difficulties in proving EDC’s effects in humans, some authors are convinced that it is impossible to conclusively demonstrate, and they would rather think in terms of prevention actions. That is why research studies should be complemented by interventions designed to reduce EDC exposure, such as primary prevention interventions, notably during pregnancy and other critical windows [28, 80, 81]. This approach also provides the scientific community and decision makers with elements possibly reinforcing their commitment to environmental health promotion.

## Trial status

Since the last recruitment took place in the end of 2019, the last visit will be planned in the end of 2020.

The choice to publish the study protocol after recruitment completion but before the last visit can be justified by the particularity of population health intervention research.

In fact, this kind of study design integrated an “interventional system” as defined by Cambon L et al. as “a set of interrelated human and non-human contextual agents within spatial and temporal boundaries generating mechanistic configurations—mechanisms—which are prerequisites for change in health” [82]. Hence, this “interventional system” required continuous adjustment to the intervention context. That is why theorization of the methodology, and consequently of the protocol, requires more time than a classic therapeutic trial. As a result, we had to step back after the intervention and integrate the dynamism of the interventional system in order to improve the quality of our assessment.

## Supplementary Information

**Additional file 1.** World Health Organization - Trial Registration Data Set

**Additional file 2.** SPIRIT checklist PREVED Study

**Additional file 3.** Study Period

## Data Availability

Paper questionnaires will be automatically anonymized to be entered on e-CRF anonymously and then stored in a locked cupboard. Biological samples will be identified only by the anonymity code. The data sets generated and analyzed during this study are not publicly available. The methodological referent and the doctoral student will have access to the final trial dataset and results will be published in a peer-reviewed journal in 2021 with a defined authorship (Additional file 3). As of now, data have yet to be analyzed.

## Abbreviations

ANOVA: Analysis of variance
BCT: Behavior change techniques
BPA: Bisphenol A
DOHaD: Developmental Origins of Health and Disease
e-CRF: Electronic case report forms
EDC: Endocrine-disrupting chemical
EDDS: Endocrine Disruptors Deux-Sèvres
ESI: Electrospray ionization
FOAD: Fetal Origins of Adult Disease
MRM: Multiple-reaction monitoring
PB: Parabens
PMI: Protection Maternelle et Infantile
POHaD: Paternal Origins of Health and Disease
WHO: World Health Organization

## References

[1] Chianese R, Troisi J, Richards S, Scafuro M, Fasano S, Guida M, Pierantoni R, Meccariello R (2018). Bisphenol A in reproduction: epigenetic effects. Curr Med Chem..

[2] Darbre PD (2017). Endocrine disruptors and obesity. Curr Obes Rep..

[3] Rivollier F, Krebs MO, Kebir O (2019). Perinatal exposure to environmental endocrine disruptors in the emergence of neurodevelopmental psychiatric diseases: a systematic review. Int J Environ Res Public Health..

[4] Von Ehrenstein OS, Ling C, Cui X, Cockburn M, Park AS, Yu F, Wu J, Ritz B. Prenatal and infant exposure to ambient pesticides and autism spectrum disorder in children: population based case-control study. BMJ. 2019 Mar 20;364:l962. doi: 10.1136/bmj.l962. Erratum in: BMJ. 2019 Jun 25;365:l4032. 10.1136/bmj.l962 PMC6425996 30894343

[5] Skogen JC, Overland S (2012). The fetal origins of adult disease: a narrative review of the epidemiological literature. JRSM Short Rep..

[6] Wadhwa PD, Buss C, Entringer S, Swanson JM (2009). Developmental origins of health and disease: brief history of the approach and current focus on epigenetic mechanisms. Semin Reprod Med..

[7] Newnham JP (2007). The developmental origins of health and disease (DOHaD) - why it is so important to those who work in fetal medicine. Ultrasound Obstet Gynecol..

[8] Soubry A (2018). POHaD: why we should study future fathers. Environ Epigenet.

[9] Wild CP (2005). Complementing the genome with an “exposome”: the outstanding challenge of environmental exposure measurement in molecular epidemiology. Cancer Epidemiol Biomarkers Prev..

[10] Wild CP (2012). The exposome: from concept to utility. Int J Epidemiol..

[11] Varshavsky J, Smith A, Wang A, Hom E, Izano M, Huang H, et al. Heightened susceptibility: a review of how pregnancy and chemical exposures influence maternal health. Reprod Toxicol. 2020 Mar;92:14–56. 10.1016/j.reprotox.2019.04.004 Epub 2019 May 2. 10.1016/j.reprotox.2019.04.004 PMC6824944 31055053

[12] World Health Organisation. Chapter 1: Executive Summary. In: Global assessment of the state-of-the-science of endocrine disruptors. http://www.who.int/ipcs/publications/new_issues/endocrine_disruptors/en/ (2015) Accessed 6 May 2021.

[13] Kabir ER, Rahman MS, Rahman I (2015). A review on endocrine disruptors and their possible impacts on human health. Environ Toxicol Pharmacol..

[14] Fenichel P, Brucker-Davis F, Chevalier N (2016). Perturbateurs endocriniens – Reproduction et cancers hormono-dépendants [Endocrine disruptors, reproduction and hormone-dependent cancers]. Presse Med..

[15] Lauretta R, Sansone A, Sansone M, Romanelli F, Appetecchia M (2019). Endocrine disrupting chemicals: effects on endocrine glands. Front Endocrinol (Lausanne).

[16] Heindel JJ, Blumberg B, Cave M, Machtinger R, Mantovani A, Mendez MA, Nadal A, Palanza P, Panzica G, Sargis R, Vandenberg LN, Vom SF (2017). Metabolism disrupting chemicals and metabolic disorders. Reprod Toxicol..

[17] Tournaire M, Epelboin S, Devouche E (2014). Diethylstilbestrol story. Therapie..

[18] Fénichel P, Brucker-Davis F, Chevalier N (2015). The history of Distilbène® (Diethylstilbestrol) told to grandchildren-the transgenerational effect. Ann Endocrinol (Paris)..

[19] Hatch EE, Troisi R, Palmer JR, Wise LA, Titus L, Strohsnitter WC, Ricker W, Hyer M, Hoover RN (2015). Prenatal diethylstilbestrol exposure and risk of obesity in adult women. J Dev Orig Health Dis..

[20] Anonymous [No authors listed]. Diethylstilbestrol (DES): also harms the third generation. Prescrire Int. 2016 Dec;25(177):294-298. PMID: 30758926. 30758926

[21] Mallozzi M, Bordi G, Garo C, Caserta D (2016). The effect of maternal exposure to endocrine disrupting chemicals on fetal and neonatal development: a review on the major concerns. Birth Defects Res C Embryo Today..

[22] Wright RO (2017). Environment, susceptibility windows, development, and child health. Curr Opin Pediatr..

[23] European Commission: Conclusions and recommendations. https://ec.europa.eu/environment/chemicals/endocrine/documents/reports_conclusions_en.htm (2016).

[24] European Commission: Communication from the commission to the european parliament, the council, the european economic and social committee and the committee of the regions. http://ec.europa.eu/transparency/regdoc/rep/1/2018/EN/COM-2018-734-F1-EN-MAIN-PART-1.PDF (2018).

[25] Gee D (2006). Late lessons from early warnings: toward realism and precaution with endocrine-disrupting substances. Environ Health Perspect..

[26] Stephenson J, Fleming TP, Godfrey KM, Barker M (2018). Preconception health - authors’ reply. Lancet..

[27] Heindel JJ, Vandenberg LN (2015). Developmental origins of health and disease: a paradigm for understanding disease cause and prevention. Curr Opin Pediatr..

[28] Grason HA, Misra DP (2009). Reducing exposure to environmental toxicants before birth: moving from risk perception to risk reduction. Public Health Rep..

[29] Fitzsimons D, Dwyer JT, Palmer C, Boyd LD. Nutrition and oral health guidelines for pregnant women, infants, and children. J Am Diet Assoc. 1998 Feb;98(2):182–6, 189 . 10.1016/S0002-8223(98)00044-3.quiz 187-8. 10.1016/S0002-8223(98)00044-3 12515420

[30] Bhutta ZA, Das JK, Rizvi A, Gaffey MF, Walker N, Horton S, Webb P, Lartey A (2013). Black RE; Lancet Nutrition Interventions Review Group, the Maternal and Child Nutrition Study Group. Evidence-based interventions for improvement of maternal and child nutrition: what can be done and at what cost?. Lancet..

[31] Koletzko B, Godfrey KM, Poston L, Szajewska H, van Goudoever JB, de Waard M, Brands B, Grivell RM, Deussen AR, Dodd JM, Patro-Golab B (2019). Zalewski BM; EarlyNutrition Project Systematic Review Group. Nutrition during pregnancy, lactation and early childhood and its implications for maternal and long-term child health: the Early Nutrition Project recommendations. Ann Nutr Metab..

[32] Siu AL (2015). U.S. Preventive Services Task Force. Screening for iron deficiency anemia and iron supplementation in pregnant women to improve maternal health and birth outcomes: U.S. Preventive Services Task Force recommendation statement. Ann Intern Med..

[33] Oliveira C, Imakawa TDS, Moisés ECD. Physical activity during pregnancy: recommendations and assessment tools. Rev Bras Ginecol Obstet. 2017 Aug;39(8):424-432. English. doi: 10.1055/s-0037-1604180. Epub 2017 Aug 7. Erratum in: Rev Bras Ginecol Obstet. 2017 Oct;39(10):584. 10.1055/s-0037-1607299 PMC10316944 29036753

[34] Brown MA, Magee LA, Kenny LC, Karumanchi SA, McCarthy FP, Saito S, Hall DR, Warren CE, Adoyi G (2018). Ishaku S; International Society for the Study of Hypertension in Pregnancy (ISSHP). Hypertensive disorders of pregnancy: ISSHP classification, diagnosis, and management recommendations for international practice. Hypertension..

[35] Sahebi Z, Kazemi A, Loripour M, Shams N (2019). An educational intervention to men for reducing environmental tobacco smoke exposure in their pregnant wives. J Matern Fetal Neonatal Med..

[36] Risica PM, Gavarkovs A, Parker DR, Jennings E, Phipps M (2017). A tailored video intervention to reduce smoking and environmental tobacco exposure during and after pregnancy: rationale, design and methods of Baby's Breath. Contemp Clin Trials..

[37] Zlatnik MG (2016). Endocrine-disrupting chemicals and reproductive health. J Midwifery Women Health..

[38] Barrett ES, Velez M, Qiu X, Chen SR (2015). Reducing prenatal phthalate exposure through maternal dietary changes: results from a pilot study. Matern Child Health J..

[39] Agence Régionale de Santé Nouvelle-Aquitaine: Regional Strategy for Environmental Health around Early Childhood [Stratégie régionale en santé environnementale autour de la petite enfance]. https://www.nouvelle-aquitaine.ars.sante.fr/strategie-regionale-en-sante-environnementale-autour-de-la-petite-enfance (2019). Accessed 6 May 2020.

[40] Bourguignon JP, Parent AS, Kleinjans JCS, Nawrot TS, Schoeters G, Van Larebeke N (2018). Rationale for Environmental Hygiene towards global protection of fetuses and young children from adverse lifestyle factors. Environ Health..

[41] Polinski KJ, Dabelea D, Hamman RF, Adgate JL, Calafat AM, Ye X, Starling AP (2018). Distribution and predictors of urinary concentrations of phthalate metabolites and phenols among pregnant women in the Healthy Start Study. Environ Res..

[42] IREPS (Instance Régionale d'Education et de Promotion de la Santé) Nouvelle-Aquitaine : Workshop 19, a pedagogical housing on environment-health. [L’atelier du 19, un logement pédagogique environnement-santé]. https://irepsna.org/actions/latelier-du-19/ (2015).

[43] Rouillon S, Deshayes-Morgand C, Enjalbert L, Rabouan S, Hardouin JB (2017). Group DisProSE, Migeot V, Albouy-Llaty M. Endocrine disruptors and pregnancy: knowledge, attitudes and prevention behaviors of French women. Int J Environ Res Public Health..

[44] Rouillon S, El Ouazzani H, Rabouan S, Migeot V, Albouy-Llaty M. Determinants of risk perception related to exposure to endocrine disruptors during pregnancy: a qualitative and quantitative study on French women. Int J Environ Res Public Health. 2018 Oct 11;15(10). pii: E2231. doi: 10.3390/ijerph15102231. 10.3390/ijerph15102231 PMC6210258 30314384

[45] Albouy-Llaty M, Rouillon S, El Ouazzani H, DisProSE G, Rabouan S, Migeot V. Environmental health knowledge, attitudes, and practices of French prenatal professionals working with a socially underprivileged population: a qualitative study. Int J Environ Res Public Health. 2019 Jul 16;16(14). pii: E2544. doi: 10.3390/ijerph16142544. 10.3390/ijerph16142544 PMC6678996 31315307

[46] Michie S, Richardson M, Johnston M, Abraham C, Francis J, Hardeman W, Eccles MP, Cane J, Wood CE (2013). The behavior change technique taxonomy (v1) of 93 hierarchically clustered techniques: building an international consensus for the reporting of behavior change interventions. Ann Behav Med..

[47] Sørensen K, Van den Broucke S, Fullam J, Doyle G, Pelikan J, Slonska Z (2012). Brand H; (HLS-EU) Consortium Health Literacy Project European. Health literacy and public health: a systematic review and integration of definitions and models. BMC Public Health..

[48] Albouy-Llaty M, Dupuis A, Grignon C, Strezlec S, Pierre F, Rabouan S, Migeot V (2015). Estimating drinking-water ingestion and dermal contact with water in a French population of pregnant women: the EDDS cohort study. J Expo Sci Environ Epidemiol..

[49] Vallieres EF, Vallerand RJ. The Rosenberg’s self-esteem scale - French-Canadian translation and validation [Traduction et validation canadienne-française de l’échelle de l’estime de soi de Rosenberg]. International Journal of Psychology. 1990;25(2):305–16. 10.1080/00207599008247865.

[50] Célant N, Guillaume S, Rochereau T. The European Health Survey - Health and Social Protection Survey 2014 [L’Enquête santé européenne - Enquête santé et protection sociale (EHIS-ESPS) 2014]. In: Les rapports de l'IRDES. https://www.irdes.fr/recherche/rapports/566-enquete-sante-europeenne-ehis-enquete-sante-et-protection-sociale-esps-2014.pdf (2014) Accessed 6 May 2020.

[51] Enquête Nationale Périnatale (ENP): Births in 2010 and their evolution since 2003 [Les naissances en 2010 et leur évolution depuis 2003). http://solidarites-sante.gouv.fr/IMG/pdf/Les_naissances_en_2010_et_leur_evolution_depuis_2003.pdf (2011). Accessed 6 May 2020.

[52] Heaman MI, Gupton AL. Psychometric testing of the Perception of Pregnancy Risk Questionnaire. Res Nurs Health. oct. 2009;32(5):493–503. 10.1002/nur.20342. 19606451

[53] Che S-R, Barrett ES, Velez M, Conn K, Heinert S, Qiu X. Using the health belief model to illustrate factors that influence risk assessment during pregnancy and implications for prenatal education about endocrine disruptors. Policy Futur Educ. 1 oct 2014;12(7):961-974.

[54] Grignon C, Venisse N, Rouillon S, Brunet B, Bacle A, Thevenot S, Migeot V, Dupuis A (2016). Ultrasensitive determination of bisphenol A and its chlorinated derivatives in urine using a high-throughput UPLC-MS/MS method. Anal Bioanal Chem..

[55] Migeot V, Dupuis A, Cariot A, Albouy-Llaty M, Pierre F, Rabouan S (2013). Bisphenol A and its chlorinated derivatives in human colostrum. Environ Sci Technol..

[56] International conference on harmonisation of technical requirements for registration of pharmaceuticals for human use. ICH Harmonised Tripartite Guideline - Validation of Analytical Procedures: Text and Methodology Q2(R1). https://database.ich.org/sites/default/files/Q2_R1__Guideline.pdf (2005). Accessed 6 May 2020.

[57] NORMAN Network: Protocol for the validation of chemical and biological monitoring methods. https://www.norman-network.net/sites/default/files/files/QA-QC%20Issues/norman_v1_v2_v3_version_02_final_feb2009.pdf (2009).

[58] European Medicines Agency, Committee for Medicinal Products for Human Use: Guideline on Bioanalytical Method Validation. https://www.ema.europa.eu/en/documents/scientific-guideline/guideline-bioanalytical-method-validation_en.pdf (2015). Accessed 6 May 2020.

[59] U.S. Department of Health and Human Services. Food and Drug Administration (FDA). Center for Drug Evaluation and Research (CDER). Center for Veterinary Medicine (CVM). Guidance for industry on bioanalytical method validation. http://www.ag-lab.org/sites/default/files/pdf/media/1/Bioanalytical%20method%20validation.pdf (2001) Accessed 6 May 2020.

[60] Glasgow RE, Vogt TM, Boles SM (1999). Evaluating the public health impact of health promotion interventions: the RE-AIM framework. Am J Public Health..

[61] Ribreau L. Environmental Health Education Workshops in the PREVED Study: Pregnancy, Prevention, Endocrine Disruptors [Ateliers d’éducation pour la santé environnementale dans le cadre de l’étude PREVED: Pregnancy, Prevention, Endocrine Disruptors]. http://nuxeo.edel.univ-poitiers.fr/nuxeo/site/esupversions/61904b1f-97b7-48a9-a234-e74ac21b9b05 (2018).

[62] Charlet NM. Prevention and promotion actions for environmental health - the example of PREVED, analysis of the action “my house, my health environment” [Les actions de prévention et de promotion en santé environnementale - L’exemple de PREVED, analyse de l’action “ma maison, mon environnement santé”]. [Master’s degree in Public Health]. Le Kremlin Bicêtre, France: Université Paris Sud; 2017.

[63] Ottawa Statement from the Sparking Solutions Summit on Population Health Intervention Research: Ottawa, Ontario, Canada April 25, 2016. Can J Public Health. 2016 Nov;107(6):e492–6. 10.17269/CJPH.107.6061 PMC6972316 30591994

[64] Jackson SF (2017). Global health promotion and population health intervention research. Glob Health Promot..

[65] Moore G, Cambon L, Michie S, Arwidson P, Ninot G, Ferron C, Potvin L, Kellou N, Charlesworth J, Alla F (2019). Discussion Panel. Population health intervention research: the place of theories. Trials.

[66] Cambon L, Alla F. Current challenges in population health intervention research. J Epidemiol Community Health. 2019 Nov;73(11):990–2. 10.1136/jech-2019-212225 Epub 2019 Jul 17. 10.1136/jech-2019-212225 31315897

[67] Rudel RA, Gray JM, Engel CL, Rawsthorne TW, Dodson RE, Ackerman JM, Rizzo J, Nudelman JL, Brody JG (2011). Food packaging and bisphenol A and bis(2-ethyhexyl) phthalate exposure: findings from a dietary intervention. Environ Health Perspect..

[68] Harley KG, Kogut K, Madrigal DS, Cardenas M, Vera IA, Meza-Alfaro G, She J, Gavin Q, Zahedi R, Bradman A, Eskenazi B, Parra KL (2016). Reducing phthalate, paraben, and phenol exposure from personal care products in adolescent girls: findings from the HERMOSA intervention study. Environ Health Perspect..

[69] Hagobian T, Smouse A, Streeter M, Wurst C, Schaffner A, Phelan S (2017). Randomized intervention trial to decrease bisphenol A urine concentrations in women: pilot study. J Womens Health (Larchmt)..

[70] Sathyanarayana S, Alcedo G, Saelens BE, Zhou C, Dills RL, Yu J, Lanphear B (2013). Unexpected results in a randomized dietary trial to reduce phthalate and bisphenol A exposures. J Expo Sci Environ Epidemiol..

[71] Fisher M, Arbuckle TE, Mallick R, LeBlanc A, Hauser R, Feeley M, Koniecki D, Ramsay T, Provencher G, Bérubé R, Walker M (2015). Bisphenol A and phthalate metabolite urinary concentrations: daily and across pregnancy variability. J Expo Sci Environ Epidemiol..

[72] Jusko TA, Shaw PA, Snijder CA, Pierik FH, Koch HM, Hauser R, Jaddoe VWV, Burdorf A, Hofman A, Tiemeier H, Longnecker MP (2014). Reproducibility of urinary bisphenol A concentrations measured during pregnancy in the Generation R Study. J Expo Sci Environ Epidemiol..

[73] Smith KW, Braun JM, Williams PL, Ehrlich S, Correia KF, Calafat AM, Ye X, Ford J, Keller M, Meeker JD, Hauser R (2012). Predictors and variability of urinary paraben concentrations in men and women, including before and during pregnancy. Environ Health Perspect..

[74] Braun JM, Smith KW, Williams PL, Calafat AM, Berry K, Ehrlich S, Hauser R (2012). Variability of urinary phthalate metabolite and bisphenol A concentrations before and during pregnancy. Environ Health Perspect..

[75] Dewalque L, Pirard C, Vandepaer S, Charlier C (2015). Temporal variability of urinary concentrations of phthalate metabolites, parabens and benzophenone-3 in a Belgian adult population. Environ Res..

[76] Lassen TH, Frederiksen H, Jensen TK, Petersen JH, Main KM, Skakkebæk NE, Jørgensen N, Kranich SK, Andersson AM (2013). Temporal variability in urinary excretion of bisphenol A and seven other phenols in spot, morning, and 24-h urine samples. Environmental Research..

[77] Nohr EA, Liew Z (2018). How to investigate and adjust for selection bias in cohort studies. Acta Obstet Gynecol Scand..

[78] Nilsen RM, Vollset SE, Gjessing HK, Skjaerven R, Melve KK, Schreuder P, Alsaker ER, Haug K, Daltveit AK, Magnus P (2009). Self-selection and bias in a large prospective pregnancy cohort in Norway. Paediatr Perinat Epidemiol..

[79] Beck E, Lechner A, Schaefer C (2017). Who seeks Teratology Information Service's advice? Assessing the risk of selection bias in observational cohort studies on drug risks in pregnancy. Reprod Toxicol..

[80] Vogel JM (2005). Perils of paradigm: Complexity, policy design, and the Endocrine Disruptor Screening Program. Environ Health..

[81] French Republic. Constitutional law n° 2005-205 of March 1, 2020 in the Charter for the Environment [Loi constitutionnelle n° 2005-205 du 1er mars 2005 relative à la Charte de l’environnement]. https://www.legifrance.gouv.fr/loda/id/JORFTEXT000000790249/ (2005) Accessed 6 May 2020.

[82] Cambon L, Terral P, Alla F (2019). From intervention to interventional system: towards greater theorization in population health intervention research. BMC Public Health..

