# Correction to: Impact of perinatal environmental health education intervention on exposure to endocrine disruptors during pregnancy—PREVED study: study protocol for a randomized controlled trial

**DOI:** 10.1186/s13063-021-05957-4

**Published:** 2021-12-29

**Authors:** Houria E. L. Ouazzani, Steeve Rouillon, Nicolas Venisse, Lynda Sifer-Rivière, Antoine Dupuis, Guillaume Cambien, Sarah Ayraud-Thevenot, Anne-Sophie Gourgues, Pascale Pierre-Eugène, Fabrice Pierre, Sylvie Rabouan, DisProSE Group, Virginie Migeot, Marion Albouy-Llaty

**Affiliations:** 1 Health-Endocrine Disruptors-EXposome (HEDEX), INSERM-CIC1402, University Hospital of Poitiers, 2 rue de la Milétrie, 86021, CEDEX Poitiers, France; 2 Faculty of Medicine and Pharmacy, University of Poitiers, 6 rue de la Milétrie, 86000 Poitiers, France; 3 BioSPharm Pole, University Hospital of Poitiers, 2 rue de la Milétrie, 86021, CEDEX Poitiers, France; 4 UMR CNRS 7285, IC2MP, Poitiers, France; 5 Research Center of Medicine, Sciences, Health and Society (Cermes 3), EHESS, University of Paris Descartes, Villejuif, France; 6 Department of Obstetrics and Gynecology and Reproductive Medicine, University Hospital of Poitiers, 2 rue de la Milétrie, 86021,CEDEX Poitiers, France

**Correction to: Trials 22, 876 (2021)**

**http://orcid.org/10.1186/s13063-021-05813-5**

Following the publication of the original article [1], we were notified of a typo in the first author’s name:

- Originally published name: Houria E. L. Ouazzani

- Corrected name: Houria El. Ouazzani

The original article has been corrected.

## References

[1] Ouazzani HEL (2021). Impact of perinatal environmental health education intervention on exposure to endocrine disruptors during pregnancy—PREVED study: study protocol for a randomized controlled trial (2021). Trials.

