# Supplementary material for: Impact of perinatal environmental health education intervention on exposure to endocrine disruptors during pregnancy—PREVED study: study protocol for a randomized controlled trial
Source: Trials. 2021 Dec 4;22:876. doi: 10.1186/s13063-021-05813-5 (PMC8642981; doi:10.1186/s13063-021-05813-5)
Supplement: Supplementary file 1 — Additional file 1. World Health Organization - Trial Registration Data Set [file 13063_2021_5813_MOESM1_ESM.pdf]

Additional File 1: World Health Organization - Trial Registration Data Set

Data Category	Information
**Primary Registry and Trial Identifying Number**	PREVED: NCT03233984
**Date of Registration in Primary Registry**	31 July 2017
**Source(s) of Monetary or Material Support**	*"Fondation de France"*
**Primary Sponsor**	University Hospital of Poitiers
**Contact for Public Queries**	Houria El Ouazzani: houria.el.fellah.el.ouazzani@univ-poitiers.fr
**Contact for Scientific Queries**	Marion Albouy-Llaty: marion.albouy-llaty@chu-poitiers.fr
**Public Title**	The PREVED Study
**Scientific Title**	Impact of perinatal environmental health education intervention on exposure to endocrine disruptors during pregnancy - PREVED study: protocol for a randomized controlled trial
**Countries of Recruitment**	FRANCE
**Health Condition(s) or Problem(s) Studied**	Exposure to endocrine disruptors
**Intervention(s)**	Three groups:<br>- A control group: Group (1)<br>- An intervention group in neutral location: Group (2)<br>- An intervention group in contextualized location: Group (3)<br>Intervention: Three workshops held between the second and the third trimesters of pregnancy on: Indoor air quality, Nutrition and Personal care products

**Key Inclusion and Exclusion Criteria**	<u>Inclusion criteria:</u><br>- pregnant women with declared pregnancy<br>- speaking French<br>- being aged 18 years or more<br>- living in the French department of Vienne and at less than 30 minutes from the contextualized space: community of agglomeration of Poitiers, ex-cantons of Saint-Georges-lès-Baillargeaux, Vouneuil-sur-Vienne, Saint-Julien-l'ars, Vouillé, Neuville, Chauvigny, La Villedieu-du-Clain, Vivonne<br>- having the intention to give birth in the maternity of the University Hospital, of the clinic "Fief de Grimoire" in Poitiers or in the Hospital of Châtellerault<br>- having signed a consent form<br><u>Exclusion criteria:</u><br>- pregnant women expecting twins or more<br>- having a complicated pregnancy<br>- not speaking French<br>- being under 18 years old or under legal protection despite being 18 or more<br>- being deprived of liberty by judicial or administrative decision<br>- undergoing psychiatric treatment<br>- not being affiliated to a social security system<br>- intending to move out during the next year<br>- having the intention to give birth in a maternity ward other than the department of Vienne<br>- being unable to express consent
**Study Type**	Open-label, monocentric, randomized controlled superiority trial
**Date of First Enrolment**	April 2017

**Sample Size**	273
**Recruitment Status**	Closed
**Primary Outcome(s)**	Percentage of participants who reported consuming manufactured or industrial food
**Key Secondary Outcomes**	Mean score of psychosocial questionnaire<br><br>Concentrations of BPA, chlorinated derivatives of bisphenol A, methyl-, ethyl-, propyl- and butyl-paraben in Urine and Colostrum<br><br>Percentage of participants who reported consuming PB-free personal care products
**Ethics Review**	Approved by the Ethics Committee of the University Hospital of Poitiers (France)<br><br>Approved by the Committee for Personal Protection (CPP) – Ouest III
**Completion date**	The end of 2020
