# Supplementary material for: Impact of perinatal environmental health education intervention on exposure to endocrine disruptors during pregnancy—PREVED study: study protocol for a randomized controlled trial
Source: Trials. 2021 Dec 4;22:876. doi: 10.1186/s13063-021-05813-5 (PMC8642981; doi:10.1186/s13063-021-05813-5)
Supplement: Supplementary file 3 — Additional file 3. Study Period [file 13063_2021_5813_MOESM3_ESM.pdf]

**Additional file 3:** Study Period

| | Closed (2019) | Ongoing | Expected in 2021 |
|---|---|---|---|
| **Enrolment** | x | | |
| **Home Visit 1: Questionnaires** | x | | |
| **Home Visit 2: Questionnaires** | x | | |
| **Home Visit 3: Questionnaires** | | x | |
| **Urine and Colostrum Analysis** | | x | |
| **Statistical Analysis** | | | x |
